# Surgical management of the aortic arch in patients with inherited aortopathy

**DOI:** 10.3389/fcvm.2022.974190

**Published:** 2022-10-20

**Authors:** Gianluca Lucchese, Rajdeep Bilkhu

**Affiliations:** Department of Cardiac Surgery, St Thomas' Hospital, London, United Kingdom

**Keywords:** aortic arch, inherited aortic disease, aortopathy, surgical management, aortic surgery

## Abstract

Surgical management of the aortic root and ascending aorta has seen an evolution over the past 50 years. Despite the widely available guidelines for management of the aortic root and ascending aorta, including in those with connective tissue disease and inherited aortopathies, there are generally no clear guideline indications for when to intervene on the aortic arch in these patients. This perhaps may be related to the fact that whilst the majority of acquired aortopathies, and also in non-syndromic aortopathies such as in bicuspid aortic valve, size criteria are utilized to decide on when to intervene, the use of size criteria may not be appropriate in those with syndromic inherited aortopathies. The aim of the present mini review is to provide a general overview and guidance for the surgical management of patients with inherited aortopathies.

## Introduction

Surgical management of the aortic root and ascending aorta has seen an evolution over the past 50 years. In 1968, Bentall et al. (1) described a method of replacement of the aortic root (including the aortic valve) and ascending aorta in what later would simply be known as the “Bentall procedure.” This technique has since been performed thousands of times for the management of patients with disease of the aortic root and ascending aorta, with excellent early and long term results (2). It remains a durable and highly reproducible technique, however, given the generally young cohort of patients with inherited aortopathy, many undergo surgery for largely prophylactic reasons; whether this is due to size criteria or due to a strong family history of aortic dissection, as described in the current international guidelines (3, 4).

Despite the widely available guidelines for management of the aortic root and ascending aorta, including in those with connective tissue disease and inherited aortopathies, there are generally no clear guideline indications for when to intervene on the aortic arch in these patients. This perhaps may be related to the fact that whilst the majority of acquired aortopathies, and also in non-syndromic aortopathies such as in bicuspid aortic valve, size criteria are utilized to decide on when to intervene, the use of size criteria may not be appropriate in those with syndromic inherited aortopathes. The increase in the number of patients suffering aortic dissection at aortic dimensions at those less than those described in the guidelines for intervention has led to many utilizing dimensions adjusted to either body surface area or height, such as the Z score, to decide on the most appropriate time to intervene on patients with aneurysmal thoracic aortic disease (5).

The aim of this review is to describe the inherited aortopathies affecting the aortic arch and the options for intervention.

## The inherited thoracic aortic aortopathies

The inherited aortopathies affecting the thoracic aorta can be broadly divided into syndromic and non-syndromic, with Marfan Syndrome, Loeys Dietz and Ehlers Danlos syndrome forming the syndromic group and those aortopathies related to bicuspid aortic valve forming the non-syndromic group.

### Marfan syndrome

Marfan syndrome is inherited in an autosomal dominant pattern and is characterized by a number of clinical features, with the majority of patients having mutations in the fibrillin-1 (FBN1) gene, which codes for the glycoprotein fibrillin-1. This forms an important component for the extracellular matrix of the aortic wall and the absence of this glycoprotein results in the reduction of elastic fiber synthesis as well as reduction in the adhesion of the elastic fibers of the aortic wall to the vascular smooth muscle cells (5). As a result there is consequent dilatation of the aortic wall.

### Loeys dietz syndrome

Loey-Dietz Syndrome is characterized by mutation affecting the TGFBR1 or TGFBR2 genes, which encode the transforming growth factors which are involved in cell signaling that promotes growth and development of body tissues which produces a variety of clinical findings including widely spaced eyes, split uvula or cleft palate, tortuous vessels in addition of course to aortic aneurysmal disease (6).

### Ehlers-danlos syndrome

Ehlers-Danlos syndrome (EDS) is characterized by skin hyperextensibility, joint hypermobility and fragility of the tissues (7). It is inherited in an autosomal dominant pattern and has a number of subtypes. Vascular EDS patients have particular problems with arterial wall fragility (5). Up to 6% of patients with vascular EDS experience either aortic dissection or rupture, at an average age of 36 years old.

### Bicuspid aortopathy

Bicuspid aortic valve (BAV) has a population prevalence of up to 2% (8). The mechanisms by which BAV leads to aortic dilatation are still being debated, however they are thought to be due both to biomechanical as well as genetic causes. A number of studies have demonstrated that by removal of the bicuspid “source,” i.e., the bicuspid aortic valve, that this may arrest dilatation of the aorta (9, 10). Despite this, there are patients who will require intervention for either a dilated or dissected ascending aorta, having had replacement of a bicuspid aortic valve previously, suggesting that a genetic cause for dilatation may also exist (11).

## Intervention for aortic arch aneurysm

### Open surgical intervention

When considering intervention for aortic arch aneurysm, there are a number of recognized approaches.

The decision for the type of surgical repair of the aortic arch is dictated by the extent of aortic arch involvement by aneurysmal disease. All types of surgical aortic arch replacement require cardiopulmonary bypass with deep hypothermia and circulatory arrest and are generally performed with replacement of the ascending aorta, with or without replacement of the aortic root. The two main approaches are as follows:

- **Hemi-aortic arch replacement:** This is whereby the aortic arch is replaced but the head and neck vessels are not replaced. The head and neck vessels, provided they are not aneurysmal or involved with aortic dissection, are preserved as a patch and a single anastomosis is performed using an obliquely beveled length of a woven polyethylene graft to the remaining thoracic aorta. This requires significant shorter circulatory arrest duration than performing total arch replacement.
- **Total aortic arch replacement:** This involves complete resection of the aortic arch, with detachment of the head and neck vessels, which can either be performed by excising an “island” of aorta containing the head and neck vessels and anastomosing this island to the vascular graft or by detaching each of the head and neck vessels separately and anastomosing these individually to a specialized vascular graft with pre-formed branches to which each of the head and neck vessels are anastomosed.

Both of these techniques allow, and are commonly performed with replacement of the proximal aorta. As well as deep hypothermia, methods of cerebral protection include retrograde cerebral perfusion, whereby oxygenated blood is passed in a retrograde fashion *via* the SVC and through the cerebral venous system, with deoxygenated being expeled from the cerebral arterial vessels. This method is particularly useful where short (<30 min) of deep hypothermia are expected for example, during hemi-aortic arch replacement. The alternative method, where longer circulatory arrest times are expected is selective antegrade cerebral perfusion, whereby oxygenated blood is passed in an antegrade fashion *via* the cerebral arterial system (12). In our practice, we routinely perform selective antegrade perfusion by passing perfusion catheters into the innominate artery and the left common carotid artery and delivering oxygenated blood *via* these routes. This provides a metabolically optimal environment for the brain during deep hypothermia and has been proven to provide excellent neuroprotection even during long circulatory arrest times (12).

### Guidelines for surgical intervention

The most recent international guidelines provide recommendations for when to intervene on the aortic root and ascending aorta, based on size criteria, and also when to intervene on the aortic arch.

The AHA and ESC published guidelines for the management of aortic disease in 2010 and 2014, respectively. Both recommend that surgery be considered for patients with isolated aortic arch dimension of >5.5 cm (3, 13). However, the guidelines are less clear on when to replace the arch when a patient is undergoing replacement of the adjacent ascending aorta. They advise that the decision to perform any arch intervention should weigh the risks of surgery with the benefits, given the increased perioperative risks.

### Intervening in inherited aortopathy

Whilst guidelines would suggest replacement of the aortic arch when the arch diameter is >5.5 cm, a number have suggested a more aggressive approach in the management of the aortic arch in those with connective tissue disease and inherited aortopathy.

Bachet et al. (14) reported on 54 patients with Marfan syndrome who had thoracic aorta replacement, some of whom had reoperative surgery after previous proximal aortic repair, and demonstrated that only 4 of 25 patients who'd had previous root replacement later required arch replacement at reoperation. They concluded that prophylactic replacement of the aortic arch in Marfan syndrome patients, at the time of proximal aortic surgery, was not indicated (14). They did however recommend a lower threshold for intervention for Marfan patients of >5 cm arch diameter, based on the fact that Marfan patients tend to dissect at lower diameters. The findings of this study were consistent with those of Schoenhoff et al. (15) who looked at 94 patient s with Marfan syndrome who underwent proximal aortic surgery for dissection as well as aneurysmal disease. They demonstrated that in those with aneurysmal disease, only 3% of patients had to undergo redo surgery and arch replacement, however this was higher in those whose initially surgery was for type A aortic dissection. They concluded again that primary prophylactic replacement of the aortic arch would not be indicated (15). Based on this, our routine practices is not to perform extensive prophylactic aortic arch replacement as we feel the addition of total arch replacement, when not strictly indicated adds to the operative complexity and on balance may not provide significant benefit. This is particularly true in the context of acute aortic dissection, whereby a more conservative approach may be more appropriate to limit the invasiveness of surgery and treat the cause of the acute dissection and in such cases this perhaps involve hemi-aortic arch replacement as opposed to total aortic arch replacement. This is consistent with the practice of other centers (16).

Loeys-Dietz has been considered a more aggressive connective tissue disease in terms of aortic dilatation and dissection and again has led groups to recommend thoracic aorta replacement at smaller diameters. In 2020, Schoenhoff et al. (17) sought to develop a strategy for the management of the arch in those with Loeys-Dietz syndrome. They compared outcomes of 79 patients with Loeys-Dietz to 256 patients with Marfan syndrome at a single center. Whilst there was no significant difference between the groups in terms of reintervention for the aortic arch in patients with aortic dissection (presumably because a portion of the aortic arch is likely to have been replaced at the initial surgery for dissection), they found that in those with Loeys-Dietz who underwent proximal aortic surgery had a greater rate of aortic arch intervention than those with Marfan syndrome (17). As a result, they recommend a more aggressive approach and suggest complete removal of the distal ascending aorta is important in patients with Loey-Dietz syndrome.

Patients with vascular Ehlers-Danlos syndrome are also at risk of dissection of the thoracic aorta at smaller diameters, however the available data is less than that for Marfan syndrome and Loeys-Dietz syndrome. The latest European and American guidelines on the management of thoracic aortic disease acknowledge this paucity of data, however both suggest surgery at aortic diameters of 4–5 cm (3, 13).

### Endovascular options for intervention

Endovascular treatments for management of the descending aorta have been widely available. TEVAR is now accepted a first line treatment in the management of descending thoracic aneurysm.

In an attempt to reduce the morbidity from open surgical repair of the aortic arch, there have been reports of endovascular repair, even to zone 0. There are two available options–fenestrated stent grafts with an orifice for the supra-aortic vessels, and branched stent grafts, which tend to be custom made (18).

Another potential hybrid solution may involve debranching of the supra-aortic vessel and implanting these on the ascending aorta, which may also be replaced. This provides a suitable landing zone for a tubular stent graft to be sited in the distal ascending aorta and excludes the aneurysmal aortic arch.

Despite this, there is a general recommendation that in patients with Marfan syndrome and other connective tissue diseases, that endovascular repair should only be considered in emergency situations or as a bridge to definitive surgical repair, given the lack of evidence to support the use of endovascular repair in these patients (13).

### Medical prevention of aortic arch dilatation

Aside from surgical and endovascular treatments of the aneurysmal aortic arch, a number of therapies to prevent or limit aortic dilatation are recognized.

In addition to general cardiovascular risk factor modification, there are therapies which may reduce or limit aortic expansion. Beta blockers should be considered as these reduced cardiac inotropy and have a consequent reduction in wall shear stress (19).

The use of angiotensin converting enzyme inhibitors and angiotensin II receptor blockers (ARBs) has been studied predominantly in the context of Marfan syndrome. The use of ARBs, particularly losartan, may be an important adjunct in downregulation of the transforming growth factor (TGF). In a small non-randomized study in a pediatric population, Brook et al. (20) concluded that the use of ARBs significantly slowed the rate of progressive aortic root dilatation.

Statin therapy has also been demonstrated in the context of abdominal aortic aneurysm to provide a protective effect by inhibiting matrix metalloproteinases. Jovin et al. (21) have demonstrated improved survival in those with thoracic aortic aneurysm who were taking a statin, up to a median of 3.6 years follow up.

Our routine practice is to ensure both patients who are under aortic surveillance, and those who have had thoracic aortic repair, remain on a beta blocker, ARB and a statin.

## Conclusions

The indications for intervention on the aortic arch remain less clear than those for proximal aortic surgery. Generally, a more aggressive approach to treatment of the aortic arch is employed when treating those with inherited aortopathies, particularly those with Loeys-Dietz syndrome and Ehlers-Danlos syndrome.

Whilst the outcomes of aortic arch surgery are improving, particularly with the advent of adjuncts such as cerebral perfusion during circulatory arrest, it still carries significant morbidity. The use of endovascular techniques in this population may not be appropriate, unless in an emergency situation. Ensuring adequate secondary prevention of aortic arch dilatation may have a role to play in limiting aortic dilatation.

## Author contributions

GL has contributed to the ideation, overview, and drafting of the manuscript. RB has contributed to the drafting and bibliographic research. Both authors contributed to the article and approved the submitted version.

## Conflict of interest

The authors declare that the research was conducted in the absence of any commercial or financial relationships that could be construed as a potential conflict of interest.

## Publisher's note

All claims expressed in this article are solely those of the authors and do not necessarily represent those of their affiliated organizations, or those of the publisher, the editors and the reviewers. Any product that may be evaluated in this article, or claim that may be made by its manufacturer, is not guaranteed or endorsed by the publisher.

## References

[1] Bentall H de Bono A. A technique for complete replacement of the ascending aorta. Thorax. (1968) 23:338. 10.1136/thx.23.4.338 5664694 PMC471799

[2] Wallen T Habertheuer A Bavaria JE Hughes GC Badhwar V Jacobs JP. Elective aortic root replacement in north america: analysis of STS adult cardiac surgery database. Ann Thorac Surg. (2019) 107:1307–12. 10.1016/j.athoracsur.2018.12.039 30685254

[3] Hiratzka LF Bakris GL Beckman JA Bersin RM Carr VF Casey Jr DE. Guidelines for the diagnosis and management of patients with thoracic aortic disease: a report of the American college of cardiology foundation/American heart association task force on practice guidelines. Circulation. (2010) 121:e266–369. 10.1161/CIR.0b013e3181d4739e 20233780

[4] Hiratzka LF Creager MA Isselbacher EM Svensson LG Nishimura RA Bonow RO. Surgery for aortic dilatation in patients with bicuspid aortic valves: a statement of clarification from the American college of cardiology/American heart association task force on clinical practice guidelines. J Am Coll Cardiol. (2016) 67:724–31. 10.1016/j.jacc.2015.11.006 26658475

[5] Fletcher AJ Syed MBJ Aitman TJ Newby DE Walker NL. Inherited thoracic aortic disease. Circulation. (2020) 141:1570–87. 10.1161/CIRCULATIONAHA.119.043756 32392100 PMC7217141

[6] Loeys BL Chen J Neptune ER Judge DP Podowski M Holm T. A syndrome of altered cardiovascular, craniofacial, neurocognitive and skeletal development caused by mutations in TGFBR1 or TGFBR2. Nat Genet. (2005) 37:275–81. 10.1038/ng1511 15731757

[7] Callewaert B Malfait F Loeys B de Paepe A. Ehlers-danlos syndromes and marfan syndrome. Best Pract Res Clin Rheumatol. (2008) 22:165–89. 10.1016/j.berh.2007.12.005 18328988

[8] Khanna AD Mahoney MS Margaryan E Topilsky Y Suri R Eidem B. Incidence of aortic complications in patients with bicuspid aortic valves. JAMA. (2011) 306:1104–13. 10.1001/jama.2011.1286 21917581

[9] Bilkhu R Youssefi P Soppa G Theodoropoulos P Phillips S Liban B. Fate of the aortic arch following surgery on the aortic root and ascending aorta in bicuspid aortic valve. Ann Thorac Surg. (2018) 106:771–6. 10.1016/j.athoracsur.2018.03.052 29698663

[10] Abdulkareem N Smelt J Jahangiri M. Bicuspid aortic valve aortopathy: genetics, pathophysiology and medical therapy. Interact Cardiovasc Thorac Surg. (2013) 17:554–9. 10.1093/icvts/ivt196 23728086 PMC3745132

[11] Girdauskas E Disha K Rouman M Espinoza A Borger MA Kuntze T. Aortic events after isolated aortic valve replacement for bicuspid aortic valve root phenotype: echocardiographic follow-up study †. Cardiothorac Surg. (2015) 48:71–6. 10.1093/ejcts/ezv259 26224339

[12] Ziganshin BA Elefteriades J. Deep hypothermic circulatory arrest. Ann Cardiothorac Surg. (2013) 2:303–15. 10.3978/j.issn.2225-319X.2013.01.05 23977599 PMC3741856

[13] Erbel R Aboyans V Boileau C Bossone E Di Bartolomeo R Eggebrecht H. 2014 ESC guidelines on the diagnosis and treatment of aortic diseases. Eur Heart J. (2014) 35:2873–926. 10.1093/eurheartj/ehu281 25173340

[14] Bachet J Larrazet F Goudot B Dreyfus G Folliguet T Laborde F. When should the aortic arch be replaced in marfan patients? Ann Thorac Surg. (2007) 83:S774–9. 10.1016/j.athoracsur.2006.10.085 17257925

[15] Schoenhoff FS Kadner A Czerny M Jungi S Meszaros K Schmidli J. Should aortic arch replacement be performed during initial surgery for aortic root aneurysm in patients with Marfan syndrome? Eur J Cardiothorac Surg. (2013) 44:346–51. 10.1093/ejcts/ezs705 23359416

[16] Westaby S Saito S Katsumata T. Acute type A dissection: conservative methods provide consistently low mortality. Ann Thorac Surg. (2002) 73:707–13. 10.1016/S0003-4975(01)03449-X 11899170

[17] Schoenhoff FS Alejo DE Black JH Crawford TC Dietz HC Grimm JC. Management of the aortic arch in patients with Loeys–Dietz syndrome. J Thorac Cardiovasc Surg. (2020) 160:1166–75. 10.1016/j.jtcvs.2019.07.130 31627951

[18] Makaloski V Tsilimparis N Rohlffs F Heidemann F Debus ES Kölbel T. Endovascular total arch replacement techniques and early results. Ann Cardiothorac Surg. (2018) 7:380. 10.21037/acs.2018.04.02 30155417 PMC6094014

[19] Groenink M de Roos A Mulder BJM Spaan JAE van der Wall EE. Changes in aortic distensibility and pulse wave velocity assessed with magnetic resonance imaging following beta-blocker therapy in the marfan syndrome. Am J Cardiol. (1998) 82:203–8. 10.1016/S0002-9149(98)00315-4 9678292

[20] Brooke BS Habashi JP Judge DP Patel N Loeys B Dietz HC. Angiotensin II blockade and aortic-root dilation in marfan's syndrome. N Engl J Med. (2008) 358:2787–95. 10.1056/NEJMoa0706585 18579813 PMC2692965

[21] Jovin IS Duggal M Ebisu K Paek H Oprea AD Tranquilli M et al. Comparison of the effect on long-term outcomes in patients with thoracic aortic aneurysms of taking versus not taking a statin drug. Am J Cardiol. (2012) 109:1050–4. 10.1016/j.amjcard.2011.11.038 22221941

